# Advantageous Detection of Significant Prostate Cancer Using a Low-Field, Office-Based MRI System

**DOI:** 10.7759/cureus.32105

**Published:** 2022-12-01

**Authors:** Selin Chiragzada, Poorvi Satya, Joseph N Macaluso, Srirama S Venkataraman, John Adams, Aleksandar N Nacev, Dinesh Kumar

**Affiliations:** 1 Biomedical Engineering, Columbia University, New York, USA; 2 Mailman School of Public Health, Columbia University, New York, USA; 3 Urology, Louisiana State University Health Foundation, New Orleans, USA; 4 Clinical Strategy, Promaxo Inc., Oakland, USA; 5 Urology, Mississippi Urology, Jackson, USA; 6 Engineering, Promaxo Inc., Oakland, USA; 7 Operations, Promaxo Inc., Oakland, USA

**Keywords:** male urology, urology, prostate biopsy, diagnosis, early detection, biopsy, cancer, prostate, mri

## Abstract

Background

Methods to diagnose prostate cancer (PCa), a highly prevalent disease, remain inadequate in terms of accuracy, cost, and logistical constraints for both patients and providers. Early and accurate detection of PCa is crucial to patient management, most notably in increasing quality of life and lowering cost burdens when considering the associated treatment and follow-up pathways. This article aims to discuss the impact to care pathways for nine patients whose PCa was detected by a novel Food and Drug Administration-cleared low-field magnetic resonance imager (MRI) for transperineal PCa interventions but was missed by standard-of-care systematic transrectal ultrasound (TRUS).

Methodology

From December 2020 to March 2022, 41 men with elevated prostate-specific antigen (PSA) levels, positive digital rectal exam findings, and Prostate Imaging Reporting & Data System scores of three or higher were enrolled. Patients first underwent targeted transperineal biopsy guided by a low-field MRI (MRIgTBx) and co-registered with T2-weighted images from a pre-procedural 3-T MRI with suspicious lesions annotated by a board-certified radiologist. Following this procedure, patients underwent standard-of-care systematic transrectal ultrasound-guided biopsy (TRUSgSBx). The entire procedure was supervised by a board-certified urologist.

Results

Of the 41 enrolled patients, both MRIgTBx and TRUSgSBx biopsies detected PCa in 20 patients. MRIgTBx detected PCa in an additional nine patients that were missed by TRUSgSBx. Five of the nine patients elected to pursue immediate treatment. Patients with suspected PCa and a negative biopsy return to the clinic every three to six months for PSA tests, with additional biopsies performed every year for cases with increasing PSA levels.

Conclusions

Early detection of PCa in nine of the 41 patients using a novel MRIgTBx method has allowed for change management resulting in an improved quality of life and cost saving for those who opted for immediate treatment. Early intervention in cases where the standard-of-care TRUSgSBx treatment was falsely negative ultimately led to a decrease in additional screening procedures, biopsies, associated tests, and an improved pathway for patient management.

## Introduction

Prostate cancer (PCa) is the most common cancer after skin cancer among men in the United States, with over 260,000 new cases diagnosed and more than 34,000 men expected to die due to PCa in 2022 [1]. Early and accurate detection of clinically significant PCa is critical in improving the overall quality of life and decreasing the cost burden for patients and the healthcare system [2]. While five-year survival rates for localized or regional PCa are above 99%, the survival rate dwindles to just 31% for cancer that has spread to other parts of the body [3]. The cost to the healthcare system for managing men with metastatic disease is much higher than early diagnosis and treatment of PCa [4]. Much has been written about the use of serum prostate-specific antigen (PSA) as a marker for cancer and its usefulness in early detection. PSA remains a first-line test for all men and especially those at risk of developing PCa due to family history, race, or other factors [5].

Direct in-bore magnetic resonance imaging (MRI)-guided biopsies demonstrate higher diagnostic accuracy from targeted sampling in comparison to both transrectal ultrasound (TRUS)-based methods such as saturation, as well as MRI-ultrasound (US) fusion biopsy methods [6,7]. For example, Demirtas et al. compared upgrading rates for patients who underwent a 12-core TRUS-guided standard prostate biopsy versus a multiparametric magnetic resonance imaging (MpMRI)-guided fusion prostate biopsy and found that the latter can provide more accurate results [6]. The costs associated with direct in-bore procedures, however, limit its utility to select academic institutions. The complexity associated with sedation is the limiting factor for performing transperineal biopsies, specifically in clinics and outpatient settings. Despite the workflow complexity the transperineal approach requires, its benefits outweigh the risk of infection associated with TRUS even in the face of antibiotic prophylaxis.

TRUS is associated with a high rate of false negatives due to its blinded approach and disproportionate targeting of the periphery of the prostate [8,9]. In recent years, MpMRI has emerged as a reliable modality to screen patients and enables the specific targeting of regions of interest that are suspicious for cancer. MpMRI has been shown to have a sensitivity of about 90% and a specificity of over 70% [10]. Combined with a targeting tool, MRI-guided biopsy has the potential to improve cancer detection rates and reduce the need for unnecessary biopsies.

A novel MRI-guided biopsy tool is used in this study. This study aims to determine the feasibility of a clinical workflow using the novel MRI to perform a needle-guided prostate biopsy. The open low-field MRI system (Promaxo, Inc., Oakland, California, United States), operating between a field strength of 58 and 74 mT, enables targeted transperineal prostate interventions under MR guidance (MRIgTBx) within a urologist’s office, thereby mitigating patient discomfort, high costs, and logistical constraints often associated with traditional MRI.

## Materials and methods

Since December 2020, the low-field MRI system has been used in a clinical study to perform prostate biopsies. In the low-field MR-guided diagnostic pathway, patients with suspected PCa due to elevated PSAs, those with positive digital rectal exam (DRE) findings, and those with Prostate Imaging Reporting & Data System (PI-RADS) scores of three or higher were enrolled in a WIRB-Copernicus Group Institutional Review Board (IRB)-approved (approval number: 20203968) study protocol at Mississippi Urology Clinic, PLLC, Jackson, Mississippi.

Following suspicion for PCa, patients are referred for a pre-procedural mpMRI acquired on a 3 T platform, performed without an endorectal coil. A radiologist interprets the mpMRI, annotates the regions of interest (ROI), and assigns them a score according to PI-RADS version 2 guidelines [9]. Note that the planning MRI technical specifications conform to PI-RADS version 2.1 recommendations. Patients with PI-RADS lesions of three or greater are eligible to undergo the MRIgTBx biopsy. During the procedure, a T2-weighted (T2W) scan of the prostate is acquired and co-registered with the pre-procedural 3 T MRI, with physical template coordinates and target depths identified and displayed on the registered images. A board-certified urologist then uses the targeted regions on the registered images as a guide and inserts needles transperineally through the template to the corresponding location and depth with the patient in a high lithotomy position. Three biopsy cores are taken from the regions suspicious for cancer previously annotated by the radiologist.

In the IRB study protocol performed at Mississippi Urology Clinic, PLLC, Jackson, Mississippi, a TRUS biopsy is performed following the low-field MR-guided biopsy procedure. As of March 2022, 41 patients have completed the study.

## Results

Patients with earlier detection

In nine out of 41 patients, cancer was missed by TRUS-guided biopsy (TRUSgSBx) but detected by MRIgTBx biopsy using the low-field MRI system. These cases are summarized in Table 1 and described in detail below. A summary of all patients and outcomes is described in Table 2.

**Table 1:** Summary of cases where transrectal ultrasound-guided biopsy (TRUSgSBx) missed and low-field MRI-guided transperineal biopsy (MRIgTBx) detected prostate cancer.

Gleason Score	Grade Group	Age	Prostate-specific antigen	Treatment pathway
4+4	4	71	8.5	Robotic prostatectomy with bilateral lymph node dissection
4+3	3	76	5.6	Brachytherapy
3+4	2	49	38.1	Robotic radical prostatectomy with left lymph node dissection
3+4	2	69	5.5	Watchful waiting
3+4	2	60	5.0	Brachytherapy seed implant
3+4	2	58	11.7	External beam therapy
3+3	1	71	12.9	External beam therapy
3+3	1	73.4	11.9	Robotic prostatectomy
3+3	1	72	9.5	Watchful waiting

**Table 2:** Summary of subjects and outcomes for all 41 patients enrolled in the study for transrectal ultrasound-guided biopsy (TRUSgSBx) and MRI-guided transperineal biopsy (MRIgTBx).

Study summary	Number of patients
Participated in MRIgTBx and TRUSgSBx	32
No cancer found in TRUSgSBx and MRIgTBx	6
Cancer detected	26
TRUSgSBx missed cancer that MRIgTBx found	9
TRUSgSBx undergraded cancer compared to MRIgTBx	3
Did not undergo the MRIgTBx procedure due to unrelated medical conditions or at the physician’s discretion	9
Total enrolled	41

In one of nine patients, a 71-year-old male with a history of elevated PSA, the MRIgTBx using the low-field MRI system found a Gleason 4+4 cancer in the posterior mid-apical region. His PSA was 8.5 ng/mL at the time of the biopsy and had three PI-RADS 4 ROIs annotated on his pre-procedural 3 T MRI scan. He elected to undergo a robotic prostatectomy with bilateral lymph node dissection. Final surgical pathology showed an even higher-grade cancer of Gleason 4+5. His PSA has been undetectable since the surgery.

Gleason Score 4+3 cancer in the right anterior region of the prostate was detected by low-field MRI biopsy in a 76-year-old male with a PSA of 5.59 ng/mL. He underwent brachytherapy about three months after his diagnosis and now has an undetectable PSA.

Four patients had 3+4 cancer detected with low-field MR-guided biopsy, with three deciding to get treatment. The first patient had an extremely high PSA of 38.1 ng/mL at 49 years old. One PI-RADS 3 and one PI-RADS 4 ROIs were targeted. The patient opted for a robotic radical prostatectomy with left lymph node dissection after his cancers were diagnosed by low-field MRI. As of March 2022, he has undergone four PSA tests following his surgery, all of which have been undetectable. Another 69-year-old patient’s PI-RADS 3 lesion was located in the right anterior periphery of the prostate with a Gleason grade of 3+4. His PSA was 5.5 ng/mL at the time of diagnosis. He has been on watchful waiting since his diagnosis in January 2021. The third patient who was 60 years old with a Gleason 3+4 diagnosis had a PSA of 5.0 ng/mL and a PI-RADS 3 lesion in the mid-right anterior section of the prostate. He underwent a brachytherapy seed implant, after which his PSA has been dropping. The last of the four patients in the 3+4 diagnosis group has had several negative biopsies and a history of elevated PSA, with the most recent being before the biopsy procedure at 11.7 ng/mL. His PI-RADS 4 lesion was located in the left anterior part. After consulting with a radiation oncologist, he received external beam therapy. He had a PSA of 3.0 ng/mL following his treatment.

In three out of nine patients, the low-field MRI found 3+3 cancer. Two of these patients decided to pursue treatment. One of them already had a low-volume 3+4 lesion detected in 2018 and was on watchful waiting. mpMRI acquired about a month before the low-field MR-guided biopsy found a PI-RADS 4 lesion in the anterior apical region. Although low risk, the patient decided to undergo external beam therapy. The other patient opted to undergo a robotic prostatectomy. Cancer was found in a PI-RADS 4 lesion located in a mid-anterior region. He has had three negative prostate biopsies and a PSA of 14.1 ng/mL. One patient is currently on watchful waiting. His PI-RADS 3 lesion was in the posterior left region.

## Discussion

The overall cancer detection rate (CDR) with the low-field MRI was found to be 71%, which is consistent with the published results for in-bore MRI biopsies. A limited number of cores are taken from the targeted region, making the procedure safer. The CDR with low-field MRI is better than the published results of TRUS and MRI-ultrasound fusion-based biopsies [11]. Additionally, the CDR from low-field is also comparable to in-bore procedures with the added benefit of being less expensive [12]. The difference in populations should be noted, however, as most of these studies had few patients with a PSA greater than 10 ng/mL [13].

In the absence of the low-field, MRI-guided biopsy, the nine patients where standard-of-care TRUSgSBx biopsy missed PCa would have delayed treatment options by a minimum of three to six months if they had not undergone targeted biopsy by some other method. As per the clinical protocol at Mississippi Urology Clinic, PLLC, Jackson, Mississippi, patients with a suspected PCa and negative biopsy will be on active surveillance, coming back every three to six months for additional PSA screening tests. If PSA levels continue rising, additional annual TRUS biopsies are performed. With the false-negative rates of up to 60% with TRUS not detecting up to 30% of clinically significant cancers, combined with potential post-biopsy complications, the cost to the healthcare system can be as high as $6,800 [14]. These costs also do not account for psychological effects which could impact not only the patient but also the family. Earlier detection with targeted procedures such as low-field MRI-guided biopsies, therefore, has allowed patients who opted for treatment to avoid the further spread of their cancer, in addition to burdens resulting from unnecessary biopsies and screening tests [15,16].

In our case, the pre-procedure 3 T mpMRI with annotations has helped limit the number of cores to three, focusing on the targets that were found to be suspicious. Additionally, the mpMRI was used for the first time to guide the needle under, intra-procedure, low-field MRI. Because the co-registration is between images from the same modality (high and ultra-low field MRI) and in the same orientation (axial), without the gland being deformed by any external probe used in ultrasound, the learning curve to localize and target the lesion should not be as steep as that between MRI and ultrasound in TRUS-fusion biopsy. Moreover, in our novel co-registered MR-MR, TBx procedure, with just three cores we were able to diagnose significant PCa lesions where TRUS biopsy missed, despite sampling 12 cores. The diagnosis of PCa by the Promaxo MRI system enabled patients to opt for a change in care management protocols.

Further, the continued advance of efforts in the field of minimally invasive focal therapy increases the need for more precise cancer lesion identification and mapping [17]. These promising new focal therapeutic approaches such as selective cryoablation, high-intensity focused ultrasound, and other technologies add an important new consideration to the role of early detection and accurate imaging. Considering the recent findings of an increased death rate from PCa the United States Preventive Services Task Force has altered its recommendations regarding the early detection of PCa [18].

The potential economic impact of delayed diagnosis

Often cited works from Yabroff and Bradley indicate a significant economic impact due to preventable cancer deaths [19]. Using a metric of the present value of lifetime earnings (PVLE), examined in different ways, it was estimated that in 2000 over $115 billion (US dollars) of total PVLE were lost due to cancer deaths. They further projected that amount to increase to over $147 billion by 2020. Lost productivity cost was greater in men ($76 billion vs. $40 billion in 2000). That was due to an increased death rate in men in 2000 as well as their increased wages versus women at that time They found that the value of life lost due to cancer death in the United States was substantial and projected to increase. That would be true even if mortality rates remained constant. Hence, they summarized that even “small decreases in mortality rates may lead to large reductions in the value of life lost.”

The debate over the impact of delayed cancer treatment is in flux as new therapies and approaches emerge. Multiple factors including patient characteristics, cancer type, treatment pathway, and other socioeconomic factors influence outcomes. All of these must be considered when compared with immediate treatment [20].

More recent studies continued to confirm the economic impact of premature loss of life. Data from the United Kingdom indicated that premature cancer deaths caused by delayed diagnoses during the initial phase of the coronavirus disease 2019 pandemic will cause significant economic losses [21].

Degeling et al. reported that over the life of PCa patients, active surveillance was not cost-effective compared with initial definitive treatment by surgery or radiation. This economic definition needs to be weighed by each patient against other factors as noted. Therefore, a modest decrease in survival shows treatment decisions for localized PCa are preference-sensitive. Patients may vary in the manner that they value avoided or delayed treatment complications versus decreased years of life [22].

## Conclusions

The results are consistent with the expected benefit of targeted MRI-guided biopsy over a systematic TRUS. The targeted MRI approach benefited the patient significantly by favorably impacting the care pathway, providing at least a six-month to one-year head-start in managing PCa.
